# Performance, Blood Lipid Profile, and the Expression of Growth Hormone Receptor (*GHR*) and Insulin-like Growth Factor-1 (*IGF-1*) Genes in Purebred and Crossbred Quail Lines

**DOI:** 10.3390/ani12101245

**Published:** 2022-05-12

**Authors:** Basant M. Shafik, Eman R. Kamel, Maha Mamdouh, Shimaa Elrafaay, Mohamed A. Nassan, Salah M. El-Bahy, Mahmoud S. El-Tarabany, Eman A. Manaa

**Affiliations:** 1 Department of Animal Wealth Development, Animal and Poultry Production, Faculty of Veterinary Medicine, Benha University, Toukh P.O. Box 13736, Qalyubia, Egypt; basant.shafik@fvtm.bu.edu.eg (B.M.S.); eman.manaa@fvtm.bu.edu.eg (E.A.M.); 2 Department of Animal Wealth Development, Economics and Farm Management, Faculty of Veterinary Medicine, Benha University, Toukh P.O. Box 13736, Qalyubia, Egypt; eman.ali@fvtm.bu.edu.eg; 3 Department of Physiology, Faculty of Veterinary Medicine, Benha University, Toukh P.O. Box 13736, Qalyubia, Egypt; maha.mamdouh@fvtm.bu.edu.eg; 4 Department of Biochemistry, Faculty of Veterinary Medicine, Benha University, Toukh P.O. Box 13736, Qalyubia, Egypt; shimaa.elrafaay@fvtm.bu.edu.eg; 5 Department of Clinical Laboratory Sciences, Turabah University College, Taif University, P.O. Box 11099, Taif 21944, Saudi Arabia; m.nassan@tu.edu.sa; 6 Department of Chemistry, Turabah University College, Taif University, P.O.Box 11099, Taif 21944, Saudi Arabia; s.elbahy@tu.edu.sa; 7 Department of Animal Wealth Development, Faculty of Veterinary Medicine, Zagazig University, Zagazig P.O. Box 44511, Sharkia, Egypt

**Keywords:** quail, performance, carcass, heterosis, IGF-1

## Abstract

**Simple Summary:**

Poultry breeding programs are established to ameliorate the genetic potential of domestic birds through a selection process and crossbreeding programs. In this context, crossbreeding has been considered as an essential tool to produce favorable genotypes, which are affected by different genetic resources and non-genetic factors. Thus, the aim was to evaluate the impact of crossing white and brown quail lines on growth performance, carcass traits, and the relative expression of growth-related genes. The crossbred WBQ quail line (male white × female brown) exhibited a significantly greater dressing percentage and better feed conversion ratio (FCR), as well as higher mRNA expression of growth hormone receptor (GHR) and insulin-like growth factor-1 (IGF-1) genes.

**Abstract:**

The aim was to evaluate the performance, blood lipid profile, and the relative expression of growth-related genes in purebred white and brown quail lines and their crossbred lines. A total of 240 one-day-old Japanese quail chicks of white and brown line, their crossbred line (WBQ: male white × female brown), and reciprocal crossbred line (BWQ: male brown × female white) were divided into four equal groups (60 birds each). The white quail line showed significantly higher final body weight, daily gain, and feed intake compared with the other quail lines (*p* < 0.001). Meanwhile, both crossbred quail lines (WBQ and BWQ) showed significantly lower FCR compared with both purebred quail lines (*p* = 0.001). Both crossbred quail lines showed greater dressing percentages compared with both purebred quail lines (*p* = 0.038). The brown quail line showed significantly (*p* = 0.05) higher levels of serum triglycerides and VLDL compared with the white and BWQ lines. The WBQ crossbred line exhibited significantly higher mRNA expression of GHR and IGF-1 genes compared with other quail lines (*p* < 0.001). Both crossbred lines (WBQ and BWQ) exhibited negative heterosis percentages for body weight (−4.39 and −3.90%, respectively) and feed intake (−10.87 and −14.59%, respectively). Meanwhile, heterosis percentages for FCR (−6.46 and −9.25%, respectively) and dressing percentage (7.54 and 6.38%, respectively) were improved in both crossbred lines. The WBQ line showed high heterosis percentages for the expression of GHR and IGF-1 genes (52.28 and 88.81%, respectively). In conclusion, the WBQ line exhibited significantly greater dressing percentage and better FCR, as well as higher mRNA expression of GHR and IGF-1 genes. These results may be helpful to improve breeding programs and to develop commercial lines of meat-type Japanese quail.

## 1. Introduction

The Japanese quail belongs to the order Galliformes, family Phasianidae, genus Coturnix, and species japonica [1]. Furthermore, the Japanese quail has been considered as the smallest avian species reared for commercial production of meat and eggs. In breeding programs, the selection for body weight (BW) in Japanese quail showed quick response [2]. The advantages of Japanese quail such as a relatively small body size, fast growth rate, short generation interval, high egg production rate, and superior meat quality have been revealed [3]. Hence, these advantages encourage genetic studies on growth performance, carcass traits, and egg production [4,5].

Poultry breeding programs are established to ameliorate the genetic potential of domestic birds through a selection process and crossbreeding programs [6]. In this context, crossbreeding has been considered as an essential tool to produce favorable genotypes that is affected by different genetic resources and non-genetic factors. Moreover, it is a good tool to combine breed effects and obtain medium performances that are preferable to the extreme values [7]. Although, reliable crossbreeding parameters including heterotic, direct, and maternal effects are required to establish a valid crossbreeding program. Moreover, comparisons among purebreds and their crossbreds are justified, depending on the large genetic variation among breeds or lines relative to genetic differences within breeds [8]. These differences are the main sources of the expected genetic potential of the breeding flocks through heterotic and complementary breed effects. It is widely accepted that there is no simple or direct explanation for heterosis. Meanwhile, it is believed that heterotic effects arise when genetically distinct individuals are crossed, depending on a dynamic diversity of mechanisms [9]. In general, heterosis results from the action of multiple loci, which affect the genetic potential of heterosis for different traits and in different hybrids. In a genome-wide association study, Abasht et al. [10] explored the genetic basis of fatness traits in two crossbred populations of meat-type broilers. They suggested that heterosis is affected by epistasis. From the subsequent results, a robust relation between heterosis and gene action is expected. However, the relation between heterotic action and specific loci should be evaluated to justify the specific gene markers for improving the crossbreeding programs [11].

It is widely accepted that growth rate is greatly influenced by heterosis, which has been utilized in the breeding strategies of domestic fowls [12]. In commercial chicken breeds, previous trials have discussed the dynamic relationship between gene expression and hybrid vigor. In this context, differential expressions of insulin-like growth factor-1 (IGF-1) gene have been reported between parental chicken lines and the F1 crossbred lines and its relevance to heterosis of meat quality traits [13]. Sun et al. [14] also demonstrated that patterns of gene expression in the liver tissues of crossbred chickens differed significantly from those of the parent lines. Although large number of genes are involved in growth performance, IGF-1 and growth hormone (GH) are the master hormones needed to maintain normal growth patterns [15]. GH usually contributes to several physiological pathways, such as growth, aging, reproduction, and egg production [16], as well as the basal metabolic rate [17]. The IGF-1 hormone has a chemical structure related to insulin and usually has several metabolic and anabolic functions [18]. Moreover, it is an essential hormone for the normal development of bone and fat tissue in fowls [19]. To the best of the authors’ knowledge, there are no previous trials that have investigated the effect of crossing on the expression of growth-related genes in quails. Hence, the aim was to evaluate the impact of crossing white and brown quail lines on growth rate, blood lipid profile, and the expression of growth hormone receptor (GHR) and IGF-1 genes.

## 2. Materials and Methods

The experiment was conducted at the Experimental Animal Research Center, Faculty of Veterinary Medicine, Benha University, Egypt. The study was conducted according to the guidelines of the Declaration of Helsinki and approved by the Animal Ethics Committee of Banha University, Egypt (approval no. BUFVTM 06-06-21).

### 2.1. Birds, Management and Experimental Design 

The parent Japanese quail lines were obtained from the Quail Production Unit, Agricultural Experiment and Research Unit, Faculty of Agriculture, Ain Shams University, Egypt. Both lines mainly differ in the colors of their feathers (white and brown). Furthermore, both lines were developed from the same random-bred population (non-selected pedigreed meat-type population), and the mating system was completely random. The random-bred base population was developed through selection for bodyweight at 28 days. A total of 240 one-day-old male Japanese quail chicks of white and brown line, their crossbred line (WBQ: male white × female brown), and reciprocal crossbred line (BWQ: male brown × female white) were the materials of this study. In addition, an excess number of sexed chicks were kept on hand to avoid any sexing problems. All chicks were of homogeneous size, and the initial body weight was 7.35 ± 0.45 g. Each genetic group was divided into 4 replicates (15 birds in each replicate). All quail chicks were reared under the same managerial and climatic conditions. All chicks were managed in an experimental room, which was supported with digital heaters to meet the required temperature and humidity. The brooding temperature was set at 35 °C for the first four days, then dropped to 32 °C at the end of the first week, and subsequently kept at 26–29 °C. The relative humidity was maintained at the recommended value (65 ± 5.0%). Feed was given ad libitum, and manual watering devices were used. Additionally, all birds were fed on the same basal diet (Table 1).

### 2.2. Body Weight, Feed Intake, and Carcass Traits 

On a weekly basis, the body weight, average daily feed intake (ADFI), and the feed conversion ratio (FCR) were recorded for each genetic group. The average daily gain was calculated as final BW-initial BW/age (days). Moreover, the overall FCR of quails from each replicate was calculated as feed intake (g)/weight gain (g). At the end of experiment (6 weeks of age), 12 birds were chosen from each genetic group (3 birds/replicate), fastened for 12 h, and then slaughtered according to the standard pre-stunning technique. Consequently, the evisceration process and removal of the internal organs were performed. Following the effective drain for 5 min, the carcasses were chilled at 2 °C for 30 min. The dressing percentage was estimated as the carcass weight relative to the live body weight [20]. Thereafter, the weight of internal organs (heart, liver, intestine, and gizzard) was recorded and illustrated as percentages of live body weight.

### 2.3. Blood Sampling and Lipid Profiles

At the end of trial, three birds were chosen from each replicate to obtain 2 ml blood samples in plain tubes, which were then centrifuged at 3000 rpm for 10 min to separate serum portion. The obtained serum samples were stored at −20 °C until analysis. Total cholesterol (TC, mg/dL), triglycerides (TG, mg/dL), and serum high-density lipoprotein (HDL, mg/dL) were measured using Centronic kits (Centronic, GmbH biological kits., Wartenberg, Germany). According to the formula of Friedewald et al. [21], serum low-density lipoprotein (LDL, mg/dL) was calculated. Moreover, the serum very-low-density lipoprotein (VLDL, mg/dL) was estimated according to the formula described by Bauer [22]. 

### 2.4. Analysis of mRNA Expression of GHR and IGF-1 Genes

For RNA analysis, three liver samples were collected from each replicate. Using the TRIZOL reagent, the RNA material was extracted (Invitrogen, Carlsbad, CA, USA). The cDNA was synthesized from RNA (cDNA Reverse Transcription kits; Applied Bio systems), and then samples of cDNA were stored at −20 °C.

For each gene, the real time-PCR products were utilized to determine the expression of these genes by using one pair of primer (Table 2). The PCR reactions were performed using the QuantiTect SYBR Green PCR Kit (Qiagen an Applied Biosystem, 7500 Fast Real- time PCR detection system). The gene expression changes of target genes were measured by the comparative 2−ΔΔCt (Ct: cycle threshold) method [23], using the housekeeping gene (β-actin) as a control.

### 2.5. Statistical Analyses

The SPSS software program (Version 16.0; IBM Corp., Armonk, NY, USA) was used to analyze the obtained data through the application of ANOVA procedures. The possible variations between the quail lines were evaluated by the Duncan multiple range test. The outputs are illustrated as means and the standard error of mean (SE). The applied model comprised the following effects:

Y_ij_ = μ + G_i_ + e_ij_ (1)

where: 

Y_ij_ = the dependent variable.

μ = the population mean.

G_i_ = the fixed effect of genetic types (i = Brown, White, WBQ and BWQ).

e_ij_ = random error.

The unit of heterosis was calculated as [(F1 − M)/M × 100%], in which M is the parental means and F1 is the crossbred means [25].

## 3. Results

### 3.1. Growh Performance and Carcass Traits 

The effects of crossing white and brown quail lines on growth performance traits are illustrated in Table 3. The white quail line showed significantly higher final body weight, ADG, and ADFI when compared with the brown quail line and both crossbred quail lines (*p* < 0.001). Meanwhile, both crossbred quail lines (WBQ and BWQ) showed significantly lower FCR compared with both purebred quail lines (*p* = 0.001).

The effects of crossing white and brown quail lines on carcass traits are described in Table 4. Both crossbred quail lines (WBQ and BWQ) showed greater dressing percentages compared with both purebred quail lines (*p* = 0.04). Meanwhile, both purebred quail lines showed higher gizzard percentages compared with both crossbred quail lines (*p* = 0.01). The current trial did not record significant differences in the heart, liver, spleen, and intestine percentages between the experimental groups.

### 3.2. Blood Lipid Profile

The effects of crossing white and brown quail lines on blood lipid profile are described in Table 5. The brown quail line showed significantly (*p* = 0.05) higher levels of serum triglycerides and VLDL compared with the white line and BWQ crossbred line. Meanwhile, the current study did not report significant differences in the levels of serum cholesterol, HDL and LDL between the experimental groups.

### 3.3. Relative Gene Expressions

**Figure 1:**
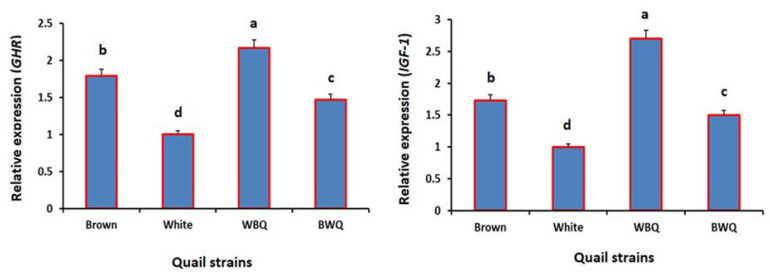
Effect of crossing white and brown quail lines on the relative expression of growth hormone receptor (GHR; *p* < 0.001) and insulin-like growth factor-1 (IGF-1; *p* < 0.001) genes. WBQ: male white × female brown quails; BWQ: male brown × female white quails; ^a,b,c,d^ values within a figure with different superscripts differ significantly.

The effect of crossing white and brown quail lines on the relative expression of *GHR* and *IGF-1* genes are illustrated in Figure 1. The WBQ crossbred line exhibited significantly higher mRNA expression of *GHR* and *IGF-1* genes compared with both purebred lines and the BWQ line (*p* < 0.001).

### 3.4. Heterosis Percentages

**Figure 2:**
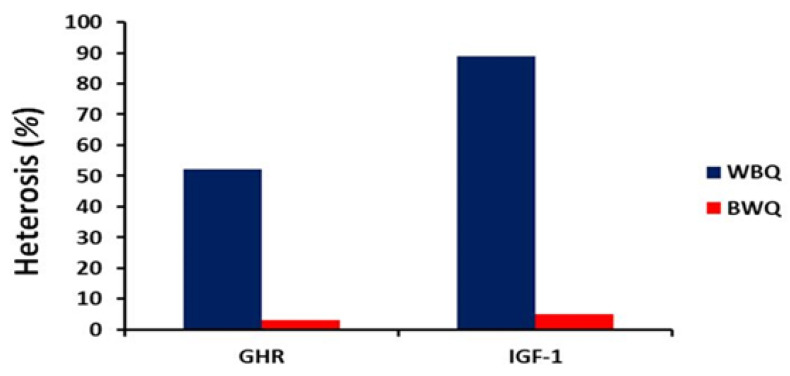
Heterosis percentages of relative expression of growth hormone receptor (GHR) and insulin-like growth factor-1 (IGF-1) genes. WBQ: male white × female brown quails; BWQ: male brown × female white quails.

Heterosis percentages of growth performance and carcass traits are described in Table 6. Both crossbred lines (WBQ and BWQ) exhibited negative heterosis percentages for body weight (−4.39 and −3.90, respectively), ADG (−4.67 and −3.86, respectively), and ADFI (−10.87 and −14.59, respectively). Meanwhile, FCR was improved in both crossbred lines (−6.46 and −9.25, respectively). Moreover, both crossbred lines exhibited positive heterosis percentages for dressing percentage (7.54 and 6.38, respectively). As illustrated in Figure 2, the WBQ line showed high and positive heterosis percentages for the expression of GHR and IGF-1 genes (52.28 and 88.81, respectively) compared with the BWQ line (3.16 and 4.90, respectively).

## 4. Discussion

Crossbreeding has been considered as a valuable tool to produce favorable genotypes, which is usually influenced by different genetic resources and non-genetic factors. Hence, the present work was conducted to elucidate the impact of crossing white and brown quail lines on growth performance, carcass traits, and the expression of some growth-related genes. In the current study, the white quail line showed significantly higher body weight, ADG, and ADFI compared with other quail lines. Consistent with these findings, Abou Khadiga et al. [26] suggested that the purebred quail lines were heavier in weight compared to their crossbred lines. Moreover, Devi et al. [27] reported significant differences among lines of Japanese quails for growth traits. In this context, the black quail line showed significantly higher body weight at the fourth week of age compared with the brown quail line [28]. In a crossbreeding experiment of two Japanese quail lines that divergently selected for body weight, Moritsu et al. [29] noticed that the weight of the crossbred lines at the fourth week of age was a little lower than the average BW of their parental lines, which almost matches the results of the current experiment.

In the current study, both crossbred quail lines (WBQ and BWQ) showed significantly lower FCR compared with both purebred quail lines. This may be attributed to the lower feed intake and averaged ADG of the crossbred lines. Additionally, Varkoohi et al. [30] suggested that selection to decrease FCR in Japanese quail increases body gain and reduces feed intake and residual feed intake as a correlated response. In agreement with these results, Jatoi et al. [31] found a significant variation in FCR among four quail lines at the fourth week of age. In a comparative study between local and imported quail lines, Akram et al. [32] reported significant differences in FCR at week four of age. Contrary to these findings, Portillo et al. [33] suggested that crossbreeding P and L quail lines did not improve the feed efficiency.

Both crossbred quail lines, herein, showed greater dressing percentages compared with both purebred quail lines. In accordance with these findings, Ali et al. [34] demonstrated significant differences among the F1 crossbred populations of brown and black Japanese quails. They found that the F1 population of the LB quail line had an overall better carcass percentage than the BL quail line. Meanwhile, others reported that quail lines of different plumage colors did not affect carcass weight and breast percentage [35]. 

In the present study, the brown quail line exhibited significantly higher levels of serum triglycerides and VLDL compared with the white and BWQ quail lines. Meanwhile, other parameters of lipogram did not differ among the quail lines. Jatoi et al. [36] reported significant differences (*p* < 0.05) in the level of serum cholesterol between imported and local Japanese quail flocks. Similarly, the plasma cholesterol levels were significantly differed among the selected lines of Japanese quails [37]. On the contrary, Rezvannejad [38] reported non-significant variations in the levels of plasma cholesterol among parental generations of Japanese quails and their crosses. 

Hormonal regulation of growth involves a complex series of interactions among different hormones including the somatotropic axis (GH, GHR, and IGF-1). Furthermore, growth hormone can affect growth directly, but the important effects are mostly mediated through the IGF-1 activity [39]. Herein, the WBQ crossbred quail line exhibited significantly higher mRNA expressions of GHR and IGF-1 genes compared with both purebred lines and the BWQ line. In this context, the WBQ line had lower ADFI and consequently better FCR compared with the purebred quail lines. This indicates that part of the recorded variation is attributed to the differences in the expression of important genes that influence different metabolic pathways. Furthermore, comparison of GHR and IGF-1 gene expression levels among the quail lines did not agree with the BW results. Perhaps the difference in BW among quail lines in this study is due to a linkage with other loci in the same chromosome region. This may explain the conflict between BW and relative gene expression in different quail lines. Indeed, the expressions of GH and IGF-1 genes had been affected by changes in the environmental conditions and feed supplements (methionine levels) during the growing period of the quails [40]. In the current experiment, all quail lines were housed under the same environmental conditions and fed the same basal diets. Thus, the observed differences in GHR and IGF-1 gene expression levels among quail lines may be attributed to the differences in the genetic makeup of each line. Consistent with these findings, Gasparino et al. [39] demonstrated that thee high-FE quail line had higher mRNA expressions of GHR and IGF-1 genes compared with the low-FE line. In a more recent study, El Sabry et al. [41] noticed that the expressions of GH and IGF-1 genes in Japanese quail lines were higher than that of European quails. On the contrary, Jia et al. [42] reported that the mRNA expressions of GHR gene were similar in the breast muscles of avian broilers and native chicken breeds at the fourth week of age. These conflicting results may indicate that the somatotropic axis genes (GHR and IGF-1) are differently regulated in different tissues and usually affected by the selection and breeding strategies.

It is believed that IGF-1 plays a crucial role in the growth rate and feed efficiency of the birds. Moreover, the lower the IGF-1 level is, the slower is the growth [43]. Additionally, IGF-1 appears to decrease the expression of genes involved in protein degradation and consequently prevents muscular atrophy and improves feed efficiency [44]. Meanwhile, previous studies have shown that high levels of GH in chickens may be associated with lower growth rates [45]. This suggests that the level of GH cannot directly explain the growth rates in chickens, and more information can be obtained by analyzing the levels of IGF-1 [46].

Although both crossbred lines (WBQ and BWQ) exhibited negative heterosis percentages for body weight and ADG, the heterosis percentages of FCR were improved. Moreover, both crossbred lines exhibited positive heterosis percentages of dressing percentage. Moritsu et al. [29] conducted a crossbreeding experiment of Japanese quail lines that divergently selected for body weight. They observed that the weight of the crossbred lines at the fourth week of age was slightly lower than the average BW of their parental lines, which is almost consistent with the results of this experiment. Piao et al. [47] also noticed that the BW of the F1 crossbred quail line (large body weight line X RR line) was intermediate between the parental lines, and the F1 crossbred line had a negative heterosis for mature body weight. In this context, crossing selected quail lines of variable body weight showed little heterosis that was negative, which may be explained as the selection for low BW being associated with major dominant genes [48] and heritability estimates of growth traits being high [49]. On the contrary, Aboul-Seoud [50] suggested that heterosis percentages for body weight at week six of age were significant, high, and positive when two selected lines of Japanese quail were crossed.

In the present study, the WBQ quail line showed high and positive heterosis percentages for GHR and IGF-1 gene expressions compared with the BWQ line. Consistent with these findings, Wang et al. [13] have reported differential expressions of IGF-1 gene between parental and F1 hybrid chickens and its relationship with the heterosis of meat quality traits. In a 4 × 4 diallel crossing experiment, Sun et al. [14] evaluated the gene expression in the liver tissues of crossbred chickens and their parents. They demonstrated that patterns of gene expression in crossbred chickens differed significantly from those of their parents. In a genome-wide association study, Abasht et al. [10] investigated the genetic basis of heterosis for a fatness-related traits in two F2 populations of meat-type broiler chickens. They also confirmed that heterosis is affected by epistasis. From the above-mentioned reports, a strong connection between heterotic effects and gene functions is evident. However, the association between heterotic effect and specific loci should be investigated to justify the specific gene markers required for improving the breeding programs [51].

## 5. Conclusions

It can be concluded that the crossbred quail lines (WBQ and BWQ) showed greater dressing percentages and better FCR compared with the purebred brown and white quail lines. Furthermore, the WBQ line exhibited a significantly higher mRNA expression of GHR and IGF-1 genes compared with the other quail lines. The WBQ exhibited positive heterosis percentages for dressing percentage and the expression of GHR and IGF-1 genes. The current study may be helpful to improve breeding programs and to develop commercial lines of meat-type Japanese quail.

## Figures and Tables

**Table 1:** Ingredients, composition, and calculated chemical analysis of the basal diets for growing quail.

Item	g/kg DM of Feed
Ingredients	
Yellow corn	556.0
Soybean meal (44%CP)	288.0
Corn gluten meal (60% CP)	105.0
Vita. and Min. mix.^†^	3.0
DL-Methionine	1.0
L-lysine	4.0
Wheat bran	20.0
Limestone	19.0
Salt (NaCl)	4.0
Calculated chemical composition (%)	
ME (kcal/kg)	2902.4
CF	3.87
CP	24.01
Na	0.17
Ca	0.82
Available phosphorus	0.41
Methionine	0.56
Lysine	1.39

^†^ Vitamin and trace mineral mixture: composition per 3 kg, Vit. A 12,000,000 I.U.; Vit. D3 2,000,000 I.U.; Vit. E 10,000 mg; Vit. K3 1000 mg; Vit. B1 1000 mg; Vit. B2 5000 mg; Vit. B6 1500 mg; Vit. B12 10 mg; niacin 30,000 mg; biotin 50 mg; folic acid 1000 mg; pantothenic acid 10,000 mg; choline chloride 500,000 mg; zinc 50,000 mg; manganese 60,000 mg; iron 30,000 mg; copper 10,000 mg; iodine 1000 mg; selenium 100 mg; cobalt 100 mg; calcium carbonate to 3 kg.

**Table 2:** Primer sequences for mRNA expression analysis.

Gene	Primer Sequences (5′-3′)	Reference	Cycle Profile
GHR	F: AACACAGATACCCAACAGCC R: AGAAGTCAGTGTTTGTCAGGG	Gasparino et al. [24]	95 °C for 10 min, 40 cycles 95 °C for 15 s (denaturation), 60 °C for 1 min (annealing and extension)
IGF-1	F: CACCTAAATCTGCACGCT R: CTTGTGGATGGCATGATCT		
β-actin	F: ACCCCAAAGCCAACAGA R: CCAGAGTCCATCACAATACC		

**Table 3:** Effect of crossing white and brown quail lines on growth performance traits.

Parameters	Quail Lines					
	Brown	White	^1^ WBQ	^2^ BWQ	^3^ SEM	*p*-Value
Final BW (g)	198 ^b^	229 ^a^	204 ^b^	205 ^b^	2.318	<0.001
^4^ ADG (g/day)	4.56 ^b^	5.28 ^a^	4.69 ^b^	4.73 ^b^	0.055	<0.001
^5^ ADFI (g/day)	17.95 ^b^	20.78 ^a^	17.23 ^c^	16.52 ^d^	1.112	<0.001
^6^ FCR	3.95 ^a^	3.94 ^a^	3.69 ^b^	3.58 ^b^	0.052	0.001

^1^ male white × female brown quails; ^2^ male brown × female white quails; ^3^ standard error of mean; ^4^ average daily gain; ^5^ average daily feed intake; ^6^ feed conversion ratio. ^a,b,c^ values within a row with different superscripts differ significantly.

**Table 4:** Effect of crossing white and brown quail lines on carcass traits.

Percentages	Quail Lines					
	Brown	White	^1^ WBQ	^2^ BWQ	^3^ SEM	*p*-Value
Dressing	65.82 ^b^	68.09 ^b^	73.62 ^a^	72.83 ^a^	2.145	0.04
Gizzard	3.03 ^a^	2.80 ^a^	2.35 ^b^	2.40 ^b^	0.108	0.01
Heart	0.83	0.78	0.85	0.86	0.055	0.85
Intestine	4.50	4.90	4.20	4.10	0.218	0.20
Liver	2.30	2.55	2.10	2.30	0.243	0.83
Spleen	0.06	0.09	0.10	0.07	0.012	0.06

^1^ male white × female brown quails; ^2^ male brown × female white quails; ^3^ standard error of mean. ^a,b^ Values within a row with different superscripts differ significantly.

**Table 5:** Effect of crossing white and brown quail lines on blood lipid profile.

Parameters	Quail Lines					
	Brown	White	^1^ WBQ	^2^ BWQ	^3^ SEM	*p*-Value
Cholesterol (mg/dL)	238.3	174.0	199.8	185.3	16.38	0.11
Triglycerides (mg/dL)	345 ^a^	240 ^b^	293 ^ab^	237 ^b^	21.2	0.05
^4^ HDL (mg/dL)	83.28	58.78	86.53	75.31	9.332	0.25
^5^ LDL (mg/dL)	86.32	67.14	54.12	62.53	8.505	0.59
^6^ VLDL (mg/dL)	69.24 ^a^	48.36 ^b^	58.62 ^ab^	47.97 ^b^	4.725	0.05

^1^ male white × female brown quails; ^2^ male brown × female white quails; ^3^ standard error of mean; ^4^ high-density lipoprotein; ^5^ low-density lipoprotein;^6^ very-low-density lipoprotein. ^a,b,ab^ values within a row with different superscripts differ significantly.

**Table 6:** Heterosis percentages of growth performance and carcass traits for crossbred quail lines.

Items	Crossbred Quails	
	^1^ WBQ	^2^ BWQ
Final BW	−4.39	−3.90
^3^ ADG	−4.67	−3.86
^4^ ADFI	−10.87	−14.59
^5^ FCR	−6.46	−9.25
Dressing %	7.54	6.38
Gizzard %	−19.52	−17.80
Heart %	4.94	6.17
Intestine %	−10.64	−12.77
Liver %	−13.58	−5.35
Spleen %	33.34	−6.67

^1^ male white × female brown quails; ^2^ male brown × female white quails; ^3^ average daily gain; ^4^ average daily feed intake; ^5^ feed conversion ratio.

## Data Availability

All data generated or analyzed during this study are included in this published paper.
